# Apoptosis Induced *via* Gamma Delta T Cell Antigen Receptor “Blocking” Antibodies: A Cautionary Tale

**DOI:** 10.3389/fimmu.2017.00776

**Published:** 2017-06-30

**Authors:** Indrani Dutta, Lynne-Marie Postovit, Gabrielle M. Siegers

**Affiliations:** ^1^Department of Oncology, University of Alberta, Edmonton, AB, Canada

**Keywords:** gamma delta T cells, blocking antibodies, cytotoxicity, mechanism, apoptosis

## Abstract

Mechanistic studies contribute greatly to our understanding of γδ T cell (γδTc) biology, aiding development of these cells as immunotherapeutic agents. The antibody blocking assay is an accepted method to determine the receptors involved in γδTc killing of tumor targets. Effectors and/or targets are preincubated with microgram quantities of monoclonal antibodies (mAb), often described by commercial sources to be useful for blocking assays. We and others have used such assays extensively in the past, correlating decreases in cytotoxicity against specific targets with involvement of the blocked receptor(s). However, we wondered whether other mechanisms might be at play beyond cytotoxicity inhibition. Indeed, administration of certain “blocking” mAb to the γδ T cell antigen receptor (γδTCR) induced γδTc death. Upon further investigation, we discovered that γδTc underwent apoptosis triggered by incubation with mAb to the γδTCR. This effect was specific, as no apoptosis was observed when αβ T cells (αβTc) were incubated with these mAb. Apoptosis was further potentiated by the presence of interleukin (IL)-2, often included in cytotoxicity assays; however, exogenous interleukin-2 (IL-2) did not contribute significantly to γδTc cytotoxicity against breast cancer cell lines. Here, we have investigated the usefulness of four mAb for use in blocking assays by assessing blocking properties in conjunction with their propensity to induce apoptosis in cultured primary human γδTc. We found that the 5A6.E9 clone was usually a better alternative to the commonly used B1 (or B1.1) and 11F2 clones; however, some variability in susceptibility to apoptosis induction was observed among donor cultures. Thus, viability assessment of primary effector cells treated with mAb alone should be undertaken in parallel with cytotoxicity assays employing blocking antibodies, to account for cytotoxicity reduction caused by effector cell death. Previous findings should be reassessed in this light.

## Introduction

γδ T cells (γδTc) are potent tumor cell killers, thought to identify their targets *via* cell surface receptors such as the γδTc antigen receptor (γδTCR) and natural killer receptors, like NKG2D (1). γδTc are particularly attractive for cancer immunotherapy, as they recognize antigens directly on transformed cells and kill quickly (with no need for priming or clonal expansion); among other advantageous features, expertly reviewed in Ref. (2), γδTc do not cause graft-versus-host disease (2). In preclinical studies, we and others have shown that γδTc kill many types of hematological and solid malignancies (2, 3). Furthermore, *in vivo* expansion of γδTc has yielded promising results in Phase I clinical trials treating metastatic prostate cancer (4), renal cell carcinoma (5), advanced breast cancer (6), and low grade non-Hodgkin lymphoma and multiple myeloma (7) reviewed together with adoptive γδTc immunotherapy trials in Ref. (8). We aim to learn more about γδTc in the context of breast cancer, to further development of γδTc immunotherapy for this disease.

Determining the mechanism(s) of action employed by γδTc against tumor cells is crucial for their further development as immunotherapy for cancer. The antibody blocking assay is an accepted method to determine the receptors involved in γδTc cytotoxicity against tumor targets (9–23). Effectors and/or targets are preincubated with microgram quantities of “blocking” monoclonal antibodies (mAb) and then co-incubated for the cytotoxicity assay, whereby decreased cytotoxicity against targets is attributed to involvement of the blocked receptor(s). A wide range of pan anti-γδTCR antibody clones have been used in these assays, including 11F2 (11, 17), B1 (14), B1.1 (9, 10, 18, 22, 23), δTCS1 (12, 21), and Immu510 (9, 10), as well as a mAb specific to the Vγ9 TCR (1, 3, 15, 16). Please note that clones B1 and B1.1 anti-γδTCR mAb clones are considered to be one and the same, simply sold by different companies (Biolegend’s Product Data Sheet for B1, Application Notes). Unfortunately, tracing the origins of commercially sold antibodies whose generation has not been documented in the literature is challenging, if not impossible.

While *bona fide* blocking of the γδTCR may indeed hinder γδTc cytotoxicity, other mechanisms, such as effector cell death, may contribute to decreases in cytotoxicity, thus leading to false interpretation of assay results. Indeed, an early study using γδTc clones showed that apoptosis can be induced by TCR/CD3 signaling in as little as 4 h incubation with soluble or immobilized 7A5 (recognizing an epitope on the Vγ9 TCR chain) or BMA030 (anti-CD3) and that this process was interleukin (IL)-2 dependent (24). To the best of our knowledge, no further studies have been undertaken to characterize other anti-γδTCR mAb in this way. We decided to test four pan anti-γδTCR mAb clones, three of which have been used previously in such blocking assays: B1 (14), B1.1 (9, 10, 18, 22, 23), and 11F2 (11, 17) plus 5A6.E9 that, to the best of our knowledge, has only been reported once in the context of γδTCR blocking in the literature (21). We set out to determine the best clone and conditions to use to further our understanding of mechanisms of γδTc cytotoxicity against tumor targets, through the correct interpretation of assay results.

## Materials and Methods

### Ethics Statement

This study was carried out in accordance with the recommendations of the Research Ethics Guidelines, Health Research Ethics Board of Alberta—Cancer Committee with written informed consent from all subjects. All subjects gave written informed consent in accordance with the Declaration of Helsinki. The protocol was approved by the Health Research Ethics Board of Alberta—Cancer Committee.

### Primary γδ T Cells

Primary human γδTc and αβTc cultures were established and maintained as described (9). Briefly, peripheral blood mononuclear cells were isolated from healthy donor blood using density gradient separation (Lymphoprep, Stem Cell Technologies, Vancouver, BC, Canada) and cultured at 1 × 10^6^ cells/ml in RPMI complete medium containing 1 μg/ml concanavalin A (Sigma-Aldrich, Oakville, ON, Canada), 10% fetal bovine serum (FBS), 1× MEM NEAA, 10 mM HEPES, 1 mM sodium pyruvate (all Invitrogen, Burlington, ON, Canada) plus 10 ng/ml recombinant human IL-2 and IL-4 (Miltenyi Biotec, Auburn, CA, USA). Every 3–4 days, cells were counted and densities adjusted to 1 × 10^6^ cells/ml by addition of fresh medium and cytokines. Cells were maintained in a humidified atmosphere at 37°C with 5% CO_2_. After 1 week, αβTc were depleted after labeling with anti-TCRαβ PE antibodies (BioLegend, San Diego, CA, USA) and anti-PE microbeads (Miltenyi Biotec), filtering through a 50 μm Cell Trics filter (Partec, Görlitz, Germany) and running through an LD depletion column (Miltenyi Biotec). The flow-through contained γδTc, which were further cultured in RPMI complete medium plus cytokines (as above) at 37°C with 5% CO_2_. For some experiments, the positively selected αβTc were also recovered and maintained. For cytotoxicity experiments, γδTc cultures were used at the end of the culture period (day 19–21), as they were most differentiated and therefore most cytotoxic at this time. Blocking assays were also typically done at this time, to mimic conditions used for cytotoxicity assays. Some experiments were done at earlier time points (days 14–16), and susceptibility to mAb-induced cell death did not appear to be significantly different, although this was not tested directly. Donor cultures are identified as follows: donor number-culture number; thus, 3-2 = the second culture derived from donor 3.

### Breast Cancer Cell Lines

Breast cancer cell lines MCF-7, T47D, and MDA-MB-231 were obtained from the American Type Culture Collection (ATCC, Manassas, VA, USA) and maintained as per ATCC guidelines.

### Calcein AM (CalAM) Labeling of Target Cells

Target cells were labeled with 5 μM CalAM as per the manufacturer’s instructions (Invitrogen/Thermo Fisher Scientific, Waltham, MA, USA). Cells were diluted to a density of 30,000 cells/100 μl medium for use in cytotoxicity assays.

### “Blocking” Antibodies

The following antihuman anti-γδTCR mAb clones were used: B1 (BioLegend, San Diego, CA, USA), B1.1 (25) (eBioscience, San Diego, CA, USA), 5A6.E9 (26) (Thermo Fisher Scientific, Waltham, MA, USA), and 11F2 (27) (Becton Dickinson, Mississauga, ON, Canada); mouse IgG (Sigma-Aldrich, Oakville, ON, Canada) was used as a control. The anti-NKG2D antibody was purchased from BioLegend (Clone 1D11). For immobilization, mAb were diluted at 1 μg/ml in PBS, then plated at 100 μl/well and incubated overnight at 4°C. Prior to blocking assays, the plates were washed twice with PBS.

### Blocking/Cytotoxicity Assay

γδTc cells were resuspended at 6 × 10^6^ cells/ml in complete medium (RPMI 1640 with 10% FBS, heat-inactivated, 1× MEM NEAA, 10 mM HEPES, 1 mM sodium pyruvate, 50 U/ml penicillin–streptomycin, and 2 mM l-glutamine—all from Invitrogen) plus 20 ng/ml recombinant human IL-2 (Miltenyi Biotec, Auburn, CA, USA) where indicated. For Fc blocking experiments, 5 μl Human TruStain FcX (BioLegend, San Diego, CA, USA) were added per 600,000 cells in 100 μl and incubated for 10 min at room temperature prior to the addition of mAb. 6 μg mAb was added to 600 μl cell suspension/test in Eppendorf tubes, then 100 μl/well plated in a 96-well round-bottomed plate. After incubation at 37°C for 30 min, 100 μl complete medium only (blocking assay only), or CalAM-labeled targets were added (cytotoxicity assay). After further incubation at 37°C for 4 h, two wells/condition of resuspended cells were pooled and stained for flow cytometry (blocking assay only). Experimental controls were untreated and IgG-treated cells. For cytotoxicity assays, plates were centrifuged and supernatants transferred to fresh 96-well plates (Falcon, U bottom, low evaporation) for CalAM fluorescence detection on a fluorimeter (FLUOstar Omega, BMG labtech). Controls were CalAM-labeled target cells incubated alone (spon = spontaneous release) and 0.05% Triton-X 100 (Thermo Fisher Scientific)-treated cells (max = maximum release). Percent lysis was calculated: [(test − spon)/(max − spon)] × 100%.

### Flow Cytometry

#### γδ T Cell Subset Identification

Cultured γδTc were stained first with 5 ng/μl Zombie Aqua (ZA) fixable viability dye (BioLegend) and then with anti-TCR Vδ1 FITC (Thermo Fisher Scientific, clone TS8.2, 1:10), anti-TCR Vδ2 PerCP (BioLegend, clone B6, 1:50), and anti-TCRγδ PE (BioLegend, clone B1, 1:25). After staining and washing, cells were fixed in FACS buffer plus 2% paraformaldehyde, stored at 4°C and analyzed by flow cytometry within 1 week. Subset and purity data for all cultures are shown in Table S1 in Supplementary Material.

#### Detection of Apoptosis (28)

Cultured γδTc were first stained with 5 ng/μl ZA fixable viability dye (BioLegend) for 15–20 min, washed with 1× annexin V (AnnV) binding buffer (BioLegend), and then stained on ice for 15 min in the dark with AnnV FITC (BioLegend, 1:20). Cells were washed and resuspended in 200 μl AnnV binding buffer plus 2% paraformaldehyde and stored at 4°C until analyzed. Flow cytometry was performed on a FACS Canto II (Becton Dickinson, Mississauga, ON, Canada), calibrated with Cytometer Set-up and Tracking beads (Becton Dickinson). Gating in forward and side scatter was done only to exclude debris; quadrant gates were set using single-stain controls. Analysis was performed using FlowJo© software (Tree Star, Ashland, OR, USA, Version 10.0.8).

### Stimulation Experiments

γδTc were incubated for 4 h at 37°C, 5% CO_2_ in serum-free and cytokine-free medium; they were then stimulated with 1 μg mAb for 1 min at 37°C. Anti-CD3 (UCHT1, BioLegend) stimulated cells were a positive control. Lysates were run on 12% SDS PAGE and Western blotting performed using a 1:400 dilution of the PathScan^®^ Multiplex Western Cocktail I (Cell Signaling Technology, Danvers, MA, USA) to detect phosphorylated signaling proteins.

### Statistics

Paired 1-tailed Student’s *t*-tests were performed (Figures 2E–G only) using Microsoft Excel version 15.3 (Microsoft, Redmond, WA, USA). ANOVA analysis followed by Bonferroni’s pairwise multiple comparison *post hoc* tests were performed using Prism 7.0 for Mac OSX (GraphPad Software, San Diego, CA, USA). Differences were considered significant when *P* < 0.05; degrees of significance are indicated by letters or asterisks as defined in the figure legends.

## Results

### An Alternate Explanation for Reduced Cytotoxicity upon Treatment with “Blocking” Antibody

**Figure 1:**
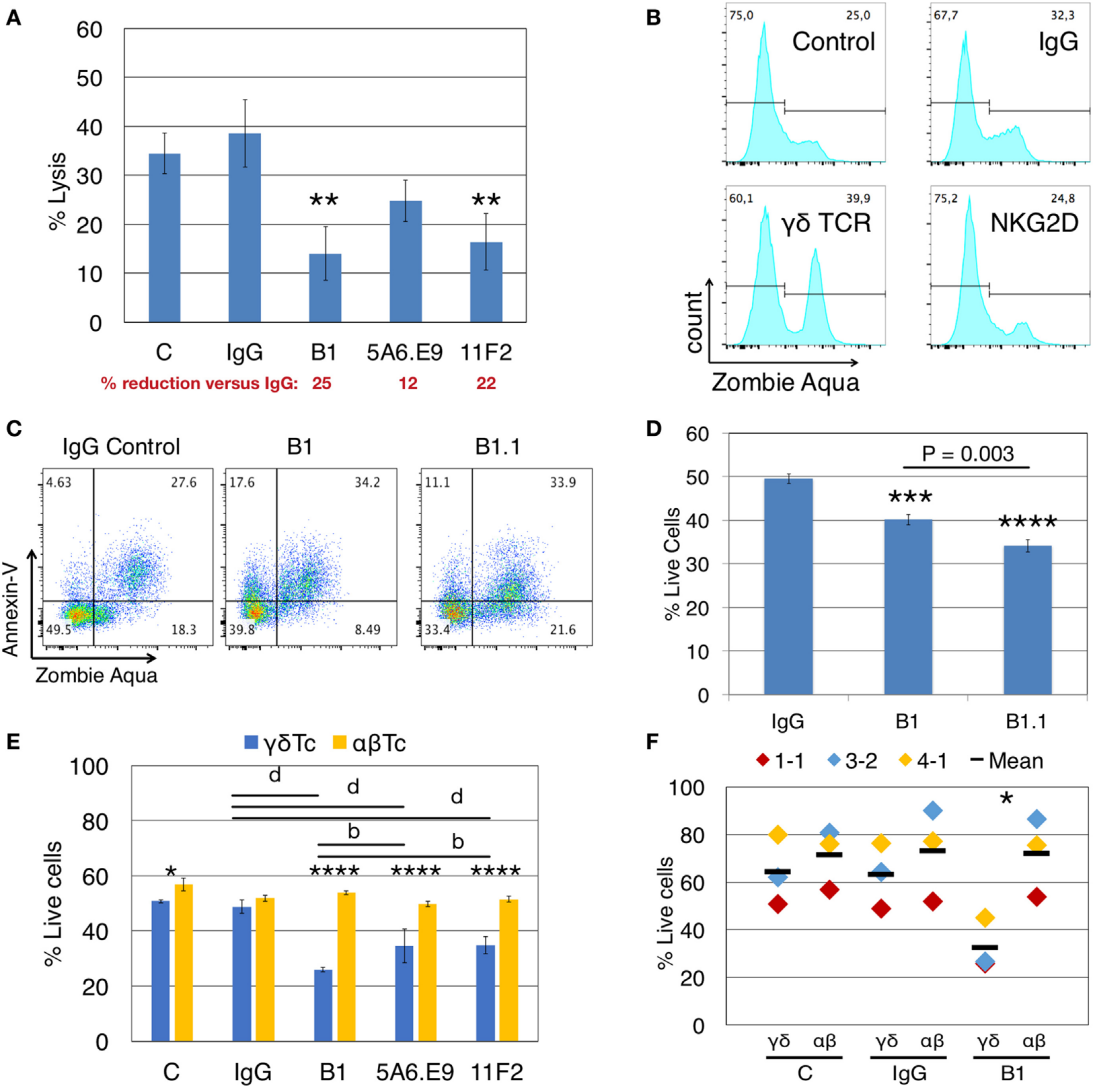
Anti-γδTCR antibodies reduce lysis of breast cancer cells concomitant with γδ T cell death. **(A)** Donor 1 day 21 primary human γδ T cells were preincubated for 30 min with (1 μg/well) or without antibodies (C = control), then incubated with calcein AM-labeled T47D targets in triplicate at a 20:1 effector:target (E:T) ratio for 4 h. A significant reduction in % lysis was observed in the presence of anti-γδTCR antibodies. Percent reduction compared to IgG (red font) was calculated: 100 − (% lysis or live cells treated with antibody/IgG × 100). **(B)** Representative example showing that treatment of 600,000 Donor 2 day 14 γδ T cells for 4.5 h with 1 μg antibody alone induces cell death as evidenced by Zombie Aqua Viability dye uptake. **(C)** Representative example indicates induction of apoptosis in Donor 3 day 21 γδ T cells *via* antibody treatment as in panel **(B)**. **(D)** Summary of results from experiment shown in panel **(C)**. **(E)** Treatment of Donor 1 αβ T cells with anti-TCRγδ antibodies does not cause cell death. **(F)** Compiled results for three independent experiments, focusing on B1 versus IgG. Error bars indicate SD (*n* = 3); significant differences compared to IgG controls **(A,D)** or between αβ and γδ T cells **(E,F)** were determined by one-way **(A,D)** or two-way **(E,F)** ANOVA followed by Bonferroni’s multiple comparisons test (*P < 0.05, **P < 0.01, ***P < 0.001, ****P < 0.0001; ^b^*P* < 0.01, ^d^*P* < 0.0001).

Incubation of γδTc with anti-human pan γδTCR mAb clones B1, 5A6.E9, and 11F2 reduces the cytotoxicity of human γδTc against T47D breast cancer cells by 1.6- to 2.8-fold/14–25% compared to IgG controls (Figure 1A). Yet if all three mAb perform an equivalent blocking function, we would expect the same decrease in lysis to occur in all cases. On the contrary, there was less reduction in average cytotoxicity with 5A6.E9 (12%) compared to 11F2 (22%) and B1 (25%) clones. One-way ANOVA followed by Bonferroni’s multiple comparisons test revealed significant differences in cytotoxicity for B1- and 11F2-blocked γδTc (*P* = 0.0023 and *P* = 0.0051, respectively), whereas the decrease in cytotoxicity with 5A6.E9 mAb was not significant (*P* = 0.1065) compared to IgG. This lead us to further investigate whether other mechanisms may be at play. Untreated controls were included in parallel to IgG and mAb treatments in most experiments, to verify similarity to IgG controls. However, statistical analyses reported herein focus on mAb versus IgG treatments; for simplicity, statistical analyses of mAb versus untreated control samples are not shown.

When we incubated γδTc alone with anti-γδTCR (B1), anti-NKG2D, or IgG control antibodies for 4.5 h, in the absence of targets, uptake of ZA viability dye indicated increased cell death in B1- but not in anti-NKG2D-treated cells (Figure 1B, gating controls in Figure S1A in Supplementary Material). Thus, the decrease in cytotoxicity observed after γδTCR “blocking” appears to be at least partially due to the untimely death of a significant proportion of effector γδTc. Importantly, this did not occur when anti-NKG2D was used, suggesting that prior interpretations implicating NKG2D in cytotoxicity were not compromised by the induction of effector cell death described here.

To further define this cell death, we extended our experiments to include AnnV staining for the detection of apoptotic cells *via* flow cytometry (28). We categorized cell death into early apoptotic (AnnV+/ZA−), late apoptotic (AnnV+/ZA+), and necrotic (AnnV−/ZA+) fractions. Treatment of γδTc with B1 and B1.1 anti-γδTCR blocking mAb resulted in increased apoptotic cell death compared to IgG controls, in both early (17.6 and 11.1 versus 4.6%) and late apoptotic compartments (34.2 and 33.9 versus 27.6%) in a representative example (Figure 1C; gating controls in Figure S1B in Supplementary Material); combined results for technical replicates from this experiment are also shown (*n* = 3, Figure 1D). One-way ANOVA followed by Bonferroni’s pairwise multiple comparison *post hoc* analysis revealed significant reductions in cell viability after incubation with B1 (*P* = 0.0003) and B1.1 (*P* < 0.0001) mAb compared to IgG. Surprisingly, a significant γδTc viability difference was also found comparing B1 and B1.1 (*P* = 0.0003), considered to be the same mAb, simply sold by different companies. The BioLegend product data sheet for B1 states “Clone B1 is also known as clone B1.1”; however, the nomenclature would suggest that B1.1 is a subclone of B1. Unfortunately, we were unable to learn anything about the generation of these clones, which is not reported in the literature. Since the effect (viability loss) was always similar in experiments carried out with both antibody clones (data not shown), yet B1.1 had a more significantly negative effect on γδTc, we used B1 for all experiments moving forward.

Induction of cell death *via* anti-γδTCR incubation was specific to γδTc, since the viability of αβTc expanded in parallel from the same donor was not compromised by incubation with B1, 5A6.E9, or 11F2 (Figure 1E). αβTc viability remained unchanged, while once again, the strongly significant reduction in γδTc viability after treatment with B1, 5A6.E9, and 11F2 compared to IgG was confirmed (*P* < 0.0001). Notably, there was also a significant difference in viability between B1 and 5A6.E9 (*P* = 0.0034) as well as between B1 and 11F2 (*P* = 0.0040), demonstrating that the detrimental effect of B1 is greater than that of the other two clones. While there was a significant difference in γδTc versus αβTc viability in the control samples, which is understandable, as these are two different cultures (albeit derived from the same donor), there was no difference in αβTc viability among antibody treatments or controls. Three independent experiments with three different donor cultures testing B1 compared to IgG and control are shown and the significant drop in γδTc viability after incubation with B1 was confirmed (Figure 1F, *P* = 0.02).

To determine whether stimulation with anti-γδTCR mAb clones differentially activates γδTc, we stimulated effector γδTc for 1 min with anti-γδTCR mAb and used anti-CD3 stimulation with the UCHT1 clone as a positive control for activation. While clones B1 and B1.1 did not cause phosphorylation of signaling proteins AKT, ERK1/2, or S6 above that of the unstimulated control, clones 11F2 and 5A6.E9 elicited a phosphorylation pattern similar to that obtained with anti-CD3 stimulation (UCHT1) suggesting active signaling (Figure S2 in Supplementary Material). Rab11 served as an internal loading control. Clones B1 and B1.1 behaved as blocking antibodies should, by not inducing activation; however, a lack of survival signaling through phosphoAKT cannot account for the cell death observed in B1-treated γδTc, since the untreated control cells in our blocking experiments did not die to the same extent, although their activation patterns are quite similar (Figure S2 in Supplementary Material, compare lanes 1–3).

### IL-2 Enhances Apoptotic Cell Death Induced by Anti-γδTCR Antibody Treatment

**Figure 2:**
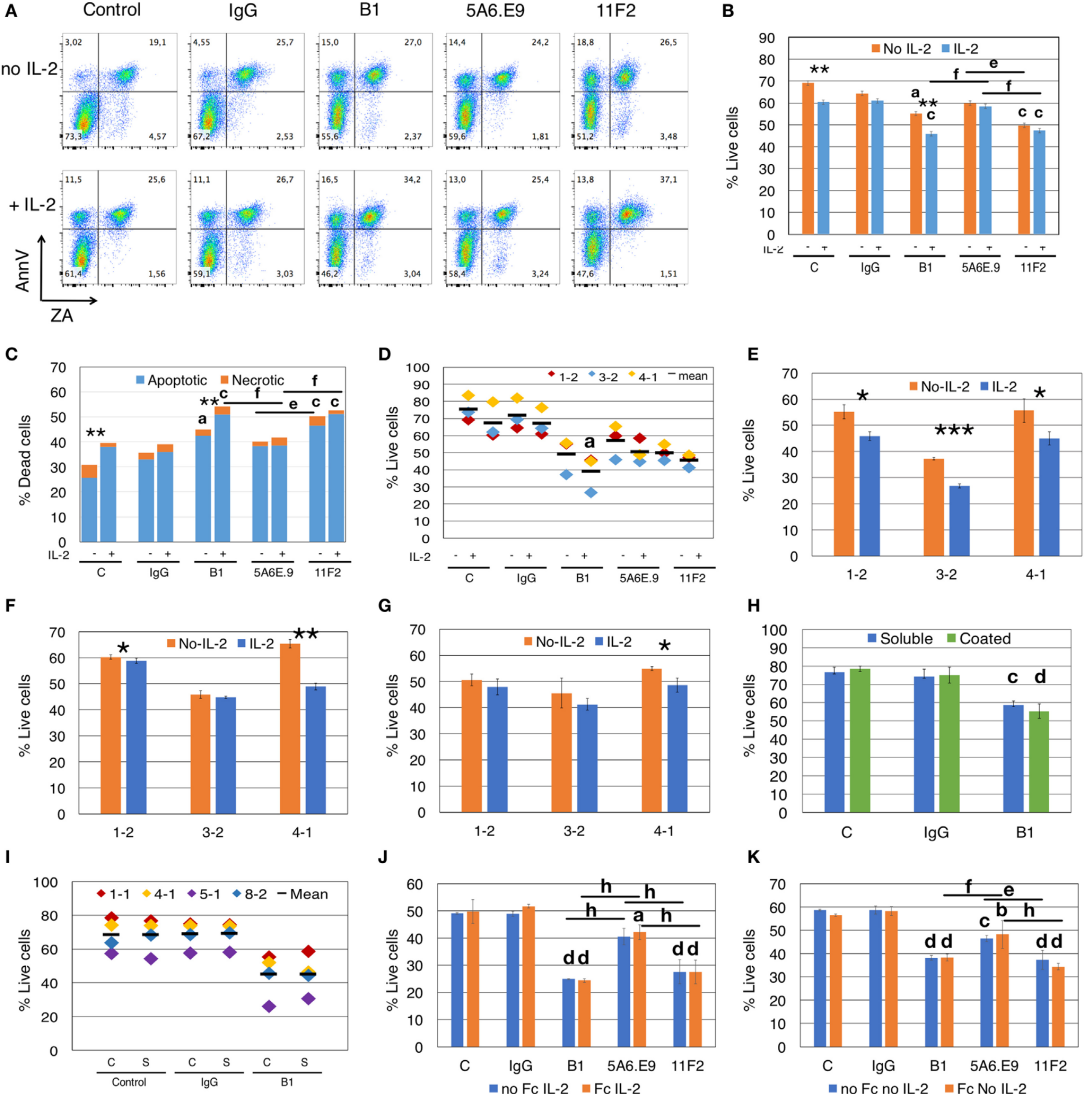
Anti-γδTCR antibodies induce apoptosis, and effects are exacerbated by interleukin-2 (IL-2). **(A)** Addition of IL-2 to blocking assays increases apoptotic cell death of γδ T cells (Donor 1, culture 2 = 1-2). Apoptotic cells are annexinV (AnnV) positive; early apoptotic cells are in the top left quadrant. Positive staining for Zombie Aqua (ZA) indicates dead cells. **(B)** For the experiment shown in panel **(A)**, % live cells are plotted for γδ T cells treated with antibodies for 4.5 h in the presence or absence of IL-2. Error bars are SD for technical replicates. This experiment is representative of three biological replicates. **(C)** Most cell death induced by antibody treatment is apoptotic cell death (blue) and is enhanced in the presence of IL-2; orange indicates necrotic cell death. The graph depicts results from the experiment shown in panels **(A,B)**. **(D)** Compiled results of experiment shown in panel **(B)** from three different donor cultures (1-2, 3-2, and 4-1). **(E)** Individual experiments in which γδTc were incubated with B1, **(F)** 5A6.E9 or **(G)** 11F2. In panels **(E–G)**, Student’s *t*-tests reveal a significant difference in cell viability in the presence or absence of IL-2; *P < 0.05, **P < 0.01, ***P < 0.001. **(H)** No differences in cell death were induced by soluble or immobilized (coated) B1 antibody treatment; however, significant cell death is observed comparing soluble B1- to IgG-treated cells. This is a representative example of three independent experiments; for this experiment, cells were from culture 1-1 on day 16. **(I)** Compiled results of experiment shown in panel **(F)** with four different donor cultures (1-1, 4-1, 5-1, and 8-2). **(J)** A representative example of two independent experiments in which γδ T cells were incubated with or without Fc blocking reagent for 10 min at room temperature prior to the addition of antibodies in the presence of IL-2. Shown here is the experiment with donor culture 8-2. **(K)** The experiment in panel **(J)** carried out in the absence of IL-2. Statistical analyses for all but panels **(E–G)** were as follows: two-way ANOVA followed by Bonferroni’s multiple comparisons tests were performed to identify significant differences between antibody-treated and IgG-treated cells (^a^*P* < 0.05; ^b^*P* < 0.01; ^c^*P* < 0.001; ^d^*P* < 0.0001) or among antibody treatments (line indicates groups compared; ^e^*P* < 0.05; ^f^*P* < 0.01; ^g^*P* < 0.001; ^h^*P* < 0.0001) as well as to determine significant viability differences in the presence or absence of IL-2 (*P < 0.05, ***P < 0.001).

We tested whether exogenous IL-2 impacts the viability of γδTc in the presence of B1, 5A6.E9, or 11F2 mAb in a 4.5 h assay. Representative dot plots show that incubation with mAb resulted in an increase in both early apoptotic (AnnV+/ZA−) and late apoptotic (AnnV+/ZA+) γδTc, even in the absence of exogenous IL-2 (Figure 2A; gating shown in Figure S1C in Supplementary Material). Two-way ANOVA followed by Bonferroni’s multiple comparison *post hoc* statistical analysis revealed significant loss of γδTc viability due to IL-2 only in control (*P* < 0.0074) and B1-treated groups (*P* = 0.0042, Figure 2B). In contrast, no IL-2-induced differences in viability were observed in cell populations treated with IgG, 5A6.E9, or 11F2 mAb; however, both B1 and 11F2 mAb decreased γδTc viability significantly compared to IgG controls. Notably, 5A6.E9 treatment did not elicit significant loss of cell viability compared to IgG in this experiment. In the presence of IL-2, 5A6.E9-treated cells were more viable than those incubated with B1 (*P* = 0.0016) or 11F2 (*P* = 0.0067); the decrease in γδTc viability after incubation with 11F2 compared to 5A6.E9 in the absence of IL-2 was also deemed significant (*P* = 0.0163). Importantly, the difference in average γδTc viability between IgG- and B1-treated cells could account for a large proportion (if not all) of the decrease in cytotoxicity associated with the use of this mAb in blocking experiments. The cell death associated with mAb incubation was mostly apoptotic cell death (Figure 2C), with significant increases in IL-2-dependent cell death observed in control and B1-treated γδTc. Statistical analysis performed on cell death data shown in Figure 2C revealed the same results as in the reciprocal live cell data in Figure 2B.

We compiled the results for three independent experiments and statistical analysis thereof confirmed significant loss of cell viability (*P* = 0.0308) in B1- but not in 11F2- or 5A6.E9-treated γδTc compared to IgG in the presence of IL-2 (Figure 2D). To further demonstrate this effect, we show the impact of IL-2 on B1-treated cells from all three experiments (Figure 2E); a synergy was observed between B1 and IL-2 that resulted in significant loss of cell viability compared to B1 treatment in the absence of IL-2. This loss was evident in all three cultures but was most pronounced in the experiment performed with Donor culture 3-2 (*P* = 0.0006). Looking more closely at 5A6.E9 treatments of these three cultures (Figure 2F) revealed a small but significant difference with IL-2 in culture 1-2 (60.3 ± 1.0 versus 58.8 ± 0.8% live cells, without or with IL-2, respectively, *P* = 0.039), and sensitivity of 4-1 to IL-2 combined with this clone (*P* = 0.0048). Culture 4-1 viability was also decreased significantly with combined 11F2 and IL-2 treatment (Figure 2G, *P* = 0.029). The full experiment done with culture 4-1 is shown in Figure S3B in Supplementary Material. No significant difference in γδTc viability was observed among the three mAb-treated groups in the presence of IL-2 (Figure S3B in Supplementary Material, *P* > 0.9999). In the absence of IL-2, 5A6.E9 elicited the least degree of γδTc cell death and was significantly better than both B1 (*P* = 0.0026) and 11F2 (*P* = 0011), which were equally detrimental (Figure S3B in Supplementary Material). Donor culture 4-1 was 78.8% Vδ2+ (Figure S3A and Table S1 in Supplementary Material), whereas 1-2 with 14.9% and 3-2 with 50.8% Vδ2+ (Table S1 in Supplementary Material) exhibited little to no difference in viability upon incubation with 5A6.E9 or 11F2, with or without IL-2 (Figures 2F,G).

Western blot analysis of cleaved Caspase 3 in total cell lysates did not reveal clear differences among IL-2-treated and untreated cells or mAb treatments over 4.5 h; however, this is a less sensitive method than flow cytometry, thus it was not surprising that we were unable to detect ~10% differences (data not shown).

We found no differences in γδTc viability when comparing the use of soluble and immobilized mAb, all in the absence of IL-2; however, B1 treatment resulted in a significant loss of viability compared to IgG-treated γδTc in both cases in the representative example shown (soluble, *P* < 0.0005; coated, *P* < 0.0001; Figure 2H). When we included 5A6.E9 and 11F2 in this experiment, again no differences in viability were observed using soluble or immobilized mAb, but significant viability losses compared to IgG were noted (Figure S4 in Supplementary Material). When we compiled results for control, IgG and B1 treatments from four independent experiments, no significant differences were identified (Figure 2I). This is likely due to inter-donor variation, suggesting that variability in cell viability among donors is greater than that observed after treatment with soluble or immobilized mAb.

We tested whether the Fc receptor might be involved in apoptosis induced by mAb treatment, but found that there were no differences in viabilities of γδTc that were or were not pretreated with an Fc blocking reagent prior to mAb incubation (Figures 2J,K). This was true whether IL-2 was present (Figure 2J) or not (Figure 2K). Significant decreases in viability were noted for mAb-treated cells (Figures 2J,K), confirming previous results (Figures 2A–I).

To understand the kinetics of apoptosis, we performed a time course experiment that revealed a highly significant decrease in live cells after 3.5 h of stimulation with B1 that extended to 4.5 h (*P* < 0.0001, Figure S5A in Supplementary Material); of note, there was a significant difference in viability, compared to IgG-treated cells, at the 30-min time point (*P* = 0.043), but this was no longer evident at 1.5 and 2.5 h post mAb treatment. A graph showing the percentage of dead cells over time is also shown (Figure S5B in Supplementary Material). Since cytotoxicity experiments are usually conducted over a minimum of 4 h, this loss of effector viability should be taken into account.

### IL-2 Is Not Required for Assessment of γδTc Cytotoxicity against Breast Tumor Targets

**Figure 3:**
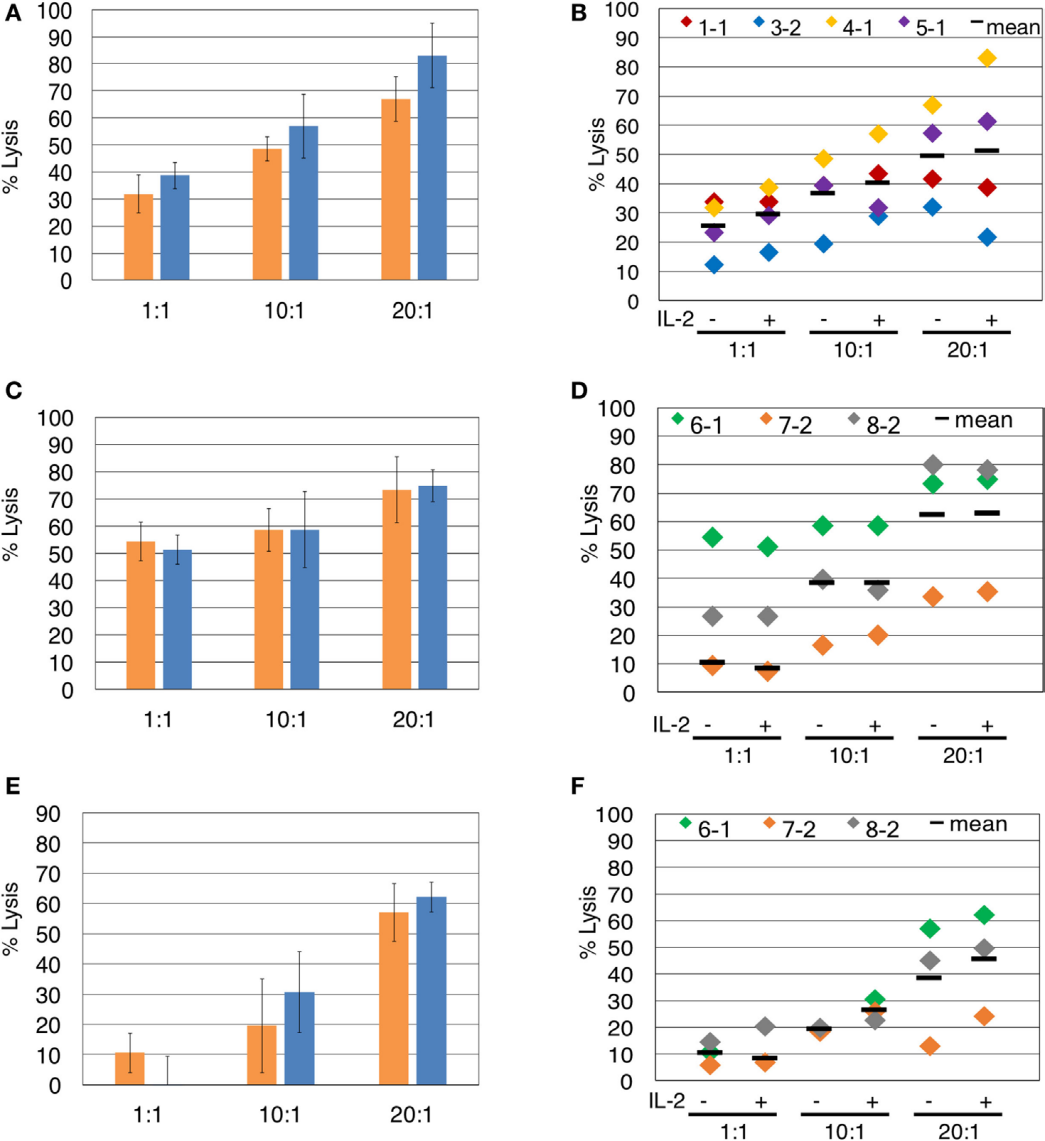
The presence of interleukin-2 (IL-2) does not significantly impact γδTc cytotoxicity against breast cancer cell lines. Comparison of γδ T cell cytotoxicity, at the indicated effector:target ratios, in the absence (orange) or presence (blue) of IL-2, against **(A)** T47D (Donor 4, culture 1, day 21 γδ T cells), representative of four independent experiments. **(B)** Compiled results of four independent cytotoxicity experiments using T47D cells as targets. **(C)** MCF-7 (Donor 6 day 19 γδ T cells), representative of three independent experiments. **(D)** Compiled results of three independent cytotoxicity experiments using MCF-7 cells as targets. **(E)** MDA-MB-231 breast cancer cell targets (Donor 6 day 19 γδ T cells). **(F)** Compiled results of three independent cytotoxicity experiments using MDA-MB-231 cells as targets. Two-way ANOVA followed by Bonferroni’s multiple comparisons analysis were performed for all experiments; no significant differences were revealed.

Since the presence of IL-2 during blocking mAb treatment caused unwanted γδTc death, we investigated whether IL-2 is necessary for assessment of γδTc cytotoxicity against breast tumor targets. In the representative example shown, IL-2 did not significantly enhance γδTc cytotoxicity against T47D at any effector:target ratio tested (Figure 3A). This was also true for the individual and compiled results of four independent experiments testing γδTc cytotoxicity against T47D (Figure 3B). We further confirmed these results in cytotoxicity assays against MCF-7 (Figures 3C,D) and MDA-MB-231 (Figures 3E,F) targets. In each case at all ratios tested, there was no significant difference in cytotoxicity of γδTc against breast tumor targets in the presence or absence of IL-2 (Figure 3).

### A Decrease in Lysis of Targets Can Be Partially Explained by Effector Cell Death upon Stimulation with Anti-γδTCR Antibodies and Thus Should Be Controlled for in γδTCR Blocking Experiments

**Figure 4:**
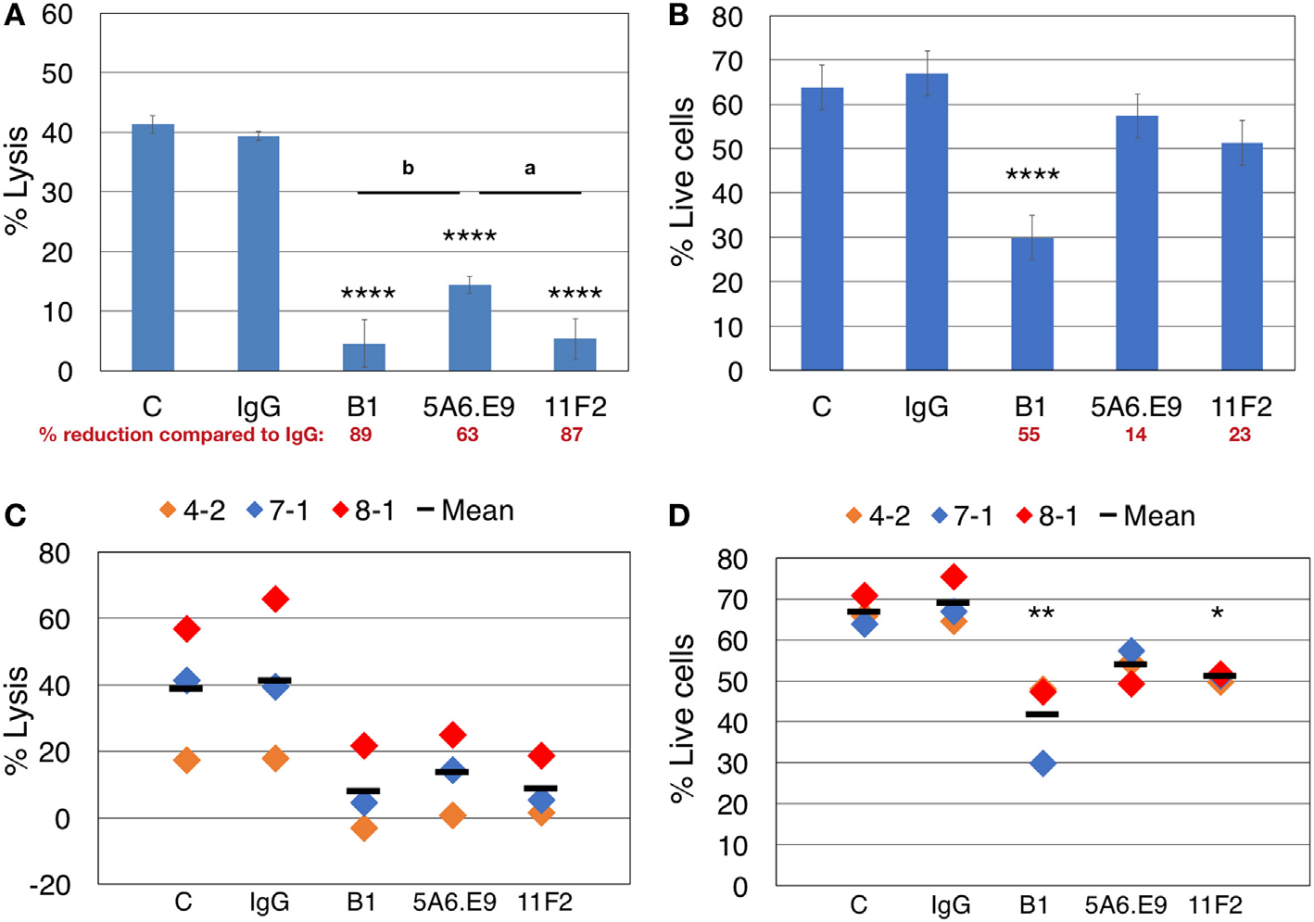
Effector blocking alone and blocking cytotoxicity assays should be performed in parallel to account for apoptosis induced by anti-γδTCR antibodies. **(A)** Donor 7 day 19 primary human γδ T cells were preincubated for 30 min with the indicated antibodies (1 μg/well) and then incubated with calcein AM-labeled targets in triplicate at a 20:1 effector:target (E:T) ratio for 4 h. **(B)** 600,000 Donor 7 day 19 γδ T cells were stimulated with 1 μg of the indicated antibodies for 4.5 h and labeled with Zombie Aqua viability dye and annexin V (AnnV) FITC; shown are the % live cells that excluded the uptake of dye and were negative for AnnV. **(C)** Compiled blocking and cytotoxicity results of three independent experiments. **(D)** Compiled blocking and viability results of three independent experiments. Panels **(A,B)** were done in parallel, as were experiments shown in panels **(C,D)**. A significant reduction in % lysis **(A,C)** or % live cells **(B,D)** was determined by one-way ANOVA followed by Bonferroni’s pairwise multiple comparison *post hoc* analysis [*P < 0.05, **P < 0.01, ****P < 0.0001 versus IgG; among antibody treatments (as indicated): ^a^*P* < 0.05; ^b^*P* < 0.01]. Percent reduction compared to IgG (in red font) was calculated: 100 − (% lysis or live cells treated with antibody/IgG × 100).

Taking all of our previous results into account, we designed parallel blocking-only and blocking plus cytotoxicity assays, in the absence of IL-2, to assess the involvement of γδTCR in killing T47D breast tumor targets, while considering anti-γδTCR mAb-induced effector cell death. Lysis of T47D targets was reduced dramatically upon treatment of γδTc with anti-γδTCR mAb; however, incubation with 5A6.E9 did not reduce lysis to quite the same extent, as significant differences in cytotoxicity observed after treatment with B1 (*P* = 0.0072) and 11F2 (*P* = 0.0128) versus 5A6.E9 were evident (Figure 4A). In line with these data, 5A6.E9 also induced significantly less cell death in these donor γδTc compared to B1, as did 11F2 (Figure 4B, both *P* < 0.0001 versus B1). Looking at the percentage of reduction in lysis or live cells compared to IgG, it is evident that at least half of the decrease in percent lysis attributed to γδTCR blocking by B1 is due to γδTc death, as opposed to blocking interactions between effectors and targets. In this experiment, 5A6.E9 and 11F2 appear to be the better clones for blocking, as γδTc viability was only reduced by 14 and 23%, respectively, compared to IgG, while they still caused 63 and 87% reductions in lysis of T47D (Figures 4A,B). A compilation of data from three independent experiments confirmed a decrease in percent lysis of T47D upon treatment with all three anti-γδTCR mAb clones; however, there was considerable variability in observed cytotoxicity of these different donor cultures against T47D breast cancer targets, rendering differences among treatment groups insignificant (Figure 4C). In contrast, significant reductions in γδTc viability remained evident, despite inter-donor variation, after treatment with B1 and 11F2, but not 5A6.E9 mAb (Figure 4D). These data confirm results shown in Figures 2A–D.

## Discussion

A reduction of γδTc cytotoxicity against tumor targets following incubation with pan γδTCR blocking mAb has been attributed to γδTCR involvement in killing (9, 11–14, 17, 21–23), yet we have discovered that certain anti-γδTCR antibodies cause γδTc apoptosis. This mechanism may be Fas dependent, suggested by the uncoupling of TCR signaling and apoptosis revealed by anti-CD3 stimulation of mature conventional T cells in wild type (B6) versus Fas-deficient (B6.lpr) mice (Fas-deficient T cells did not undergo apoptosis) (29).

Gan and colleagues also described Fas-dependent apoptosis of Daudi activated but not naïve human γδTc stimulated with 5A6.E9 in the context of magnetic cell separation (30). While Fas was present throughout, surface expression of FasL on restimulated cells was most pronounced at 8 h poststimulation, suggesting that an incubation time of 4 h for a cytotoxicity assay is appropriate to avoid even more pronounced effector cell death (30). Our kinetics experiment with B1-treated γδTc showed that appreciable cell death was first detectable between 3.5 and 4.5 h (Figure S5 in Supplementary Material), supporting even shorter incubation times. In line with most of our results with 5A6.E9 (Figures 2A–D,F), the presence or absence of IL-2 in culture after stimulation did not alter the rate or extent of γδTc cell death (30).

We typically include exogenous IL-2 in our cytotoxicity assays (Figure 1), since IL-2 is thought to enhance the cytotoxic potential of γδTc. While not inducing cytotoxicity on its own, IL-2 increased Vδ2 γδTc lysis of Daudi Burkitt’s lymphoma and TU167 squamous carcinoma cell lines in a dose-dependent manner in the presence of tumor cells or phosphoantigen (31). Janssen and colleagues determined that the apoptotic effect of TCR stimulation was IL-2 dependent (24); such synergy was also evident in γδTc treated with the B1 anti-γδTCR clone in the presence of IL-2 (Figure 2). In published reports of *in vitro* γδTc cytotoxicity against breast cancer cell lines, it is unclear whether IL-2 was included in the assays (20, 32). Our data suggest that exogenous IL-2 is unnecessary for the assessment of γδTc cytotoxicity against breast cancer cell lines (Figure 3) and indeed its propensity to drive activated T cells into apoptosis (33) further warrants its exclusion from such assays.

Our experiments have revealed that the 5A6.E9 clone is a better mAb to “block” the γδTCR than B1, as it typically induced the least amount of apoptosis and appeared not to synergize with IL-2 to enhance this unwanted effect; however, this behavior was somewhat donor- and likely γδTc subset dependent (Figures 2A–D,F), further discussed below.

We admittedly did not screen every available anti-γδTCR antibody in our assays, but rather sought to document this unwanted effect to encourage investigators to consider this issue when performing their own blocking experiments. It is possible that primary γδTc generated using other protocols may be more or less sensitive to apoptosis induction *via* B1 or 11F2. Thus, it will be important for researchers to test blocking mAb on γδTc cultured using their own protocols. For example, Guo et al.’s apoptosis-resistant γδTc (34) may be less susceptible to B1-induced apoptosis than γδTc cultured in our lab (9). In a later study with these cells, it is unclear which anti-γδTCR clone was used for blocking (20); however, the best way to assess γδTc susceptibility to apoptosis upon anti-γδTCR treatment would have been to perform blocking assays on γδTc alone and assess viability in parallel with cytotoxicity assays, as we have done here (Figure 4).

We did not test V-segment-specific mAb in our study; however, Janssen and colleagues have shown that Vγ9-specific mAb 7A5 induced apoptosis in γδTc clones, potentiated by addition of IL-2 (24). The extent of apoptosis was the same with soluble or immobilized mAb, as we also observed with B1 (Figures 2H,I). It could be informative to determine whether other V-segment-specific mAb induce γδTc apoptosis; if researchers propose to employ these mAb in blocking experiments, then it would indeed be important to test for this effect in advance. If apoptosis induction is evident, these mAb may be employed for specific *in vivo* elimination of these cells. Furthermore, if we could map the epitopes recognized by pan-γδTCR and V-segment-specific mAb that induce apoptosis in γδTc, perhaps small molecules could be designed to induce apoptotic signaling pathways and thereby deplete specific γδTc subsets implicated in various pathologies *in vivo*.

Controversy over the effectiveness of *in vivo* depletion strategies was addressed by Koenecke and colleagues, who determined that injection of anti-γδTCR mAb into mice did not delete γδTc as expected, but rather caused receptor internalization, rendering the cells “invisible” (35). Kinetics experiments revealed, however, that ~10–20% of γδTc were indeed lost as of 14 days; the authors attributed this to activation-induced γδTc death (35), which is what we observed here on a much shorter time scale *in vitro*. While GL3 and UC7-13D5 mAb recognize at least partially overlapping epitopes, the higher affinity of GL3 led to greater TCR internalization (35), confirming that even mAb recognizing similar structures have differential effects and should therefore be tested and chosen wisely.

In our hands, in separate experiments, B1 mAb stimulation typically resulted in an average γδTc viability loss of ~10–15% (Figures 1D and 2B–G). This was in line with cytotoxicity differences observed comparing blocking with B1 to 5A6.E9 (38.6 − 24.8% = 13.8%), suggesting that roughly half of the dramatic “blocking” effect observed upon B1 incubation can be attributed to unintended induction of apoptosis in γδTc. While in assays done in parallel these numbers were higher (Figure 4), the estimate of a ~50% reduction attributable to B1-induced cell death still held true. Importantly, our results with 5A6.E9 suggest that the γδTCR is indeed involved in γδTc killing of breast cancer targets.

While we did not directly address the susceptibility of individual γδTc subsets to apoptosis induction *via* anti-γδTCR antibodies, we might assume that the Vδ2 subset was most strongly affected, due to this subset’s documented sensitivity to activation-induced cell death (36–38). Donor culture 4-1 was mainly Vδ2 (78.8%, Figure S3A and Table S1 in Supplementary Material) and susceptible to enhanced apoptosis through IL-2 in combination with each of the three tested anti-TCRγδ mAb (Figures 2E–G; Figure S3B in Supplementary Material). Note that Student’s *t*-tests were employed to assess significant differences in IL-2-treated versus -untreated cells in Figures 2E–G, but that ANOVA was used for the full experiment in Figure S3B in Supplementary Material, explaining differences in statistical outcomes for 11F2-treated samples (compare Figure 2G and Figure S3B in Supplementary Material). Cultures comprising lower percentages of Vδ2 cells (1-2 and 3-2, Table S1 in Supplementary Material) exhibited little to no difference in viability upon incubation with 5A6.E9 or 11F2, with or without IL-2 (Figures 2F,G). These data suggest that Vδ2 is more sensitive to apoptosis induced by anti-TCRγδ mAb in the presence of IL-2. Since most studies focus on the Vδ2 subset, it is crucial that researchers take this unintended effect into account.

Previous blocking assays reported in the literature have potentially unwittingly overstated γδTCR involvement in lysis of target cells by assuming that decreased target death is due to blocking of the TCR, not realizing that a proportion of effector γδTc may have undergone apoptosis. If, for example, anti-TCRVδ1 mAb clones induce γδTc apoptosis, then the TCR may not be involved in Vδ1 γδTc killing of MEC1 leukemia cells since B1 and anti-Vδ1 both appeared to “block” MEC1 lysis to a similar extent (14). In our own previous work, Immu510 and B1.1 clones decreased percent lysis of PC-3M prostate cancer cells to the same extent, suggesting that Immu510 may be as detrimental to γδTc viability as B1.1 (9). In that experiment, using a Vδ2-predominant culture (77.4% Vδ2, 14.2% Vδ1), neither anti-Vδ1 (TS8.2) nor anti-Vδ2 (B6) clones significantly reduced cytotoxicity against PC-3M, suggesting that the TCR was not involved (9). In another experiment with a Vδ1 predominant γδTc culture (55.1% Vδ1, 11.7% Vδ2), derived from a different donor, anti-Vδ1 did decrease lysis significantly, yet this may have been due to loss of γδTc as opposed to bona fide Vδ1 TCR blocking (9), although Vδ1 may be less susceptible to apoptosis induction than Vδ2, as discussed above. In contrast, a lack of decrease in tumor cell death after mAb treatment suggests that the γδTc employed were not susceptible to mAb-induced apoptosis. For example, Vδ1 γδTc expressing natural cytotoxicity receptors were not susceptible to apoptosis induced by B1.1 or Immu510, as no drop in MOLT4 lysis was observed (10). Likewise, blocking with B1.1 and Immu510 had no influence on Vδ2 γδTc cytotoxicity against Jurkat or Molt4 (18). Thus, the TCR was not implicated in cytotoxicity and no false misinterpretation resulted (10, 18). Yet γδTCR involvement in killing of ULBP4-overexpressing murine thymoma EL4 (22) as well as hMSH2-expressing HeLa cells (23) may have been overstated, if the γδTc employed in these studies were as susceptible to B1.1-mediated apoptosis as ours were.

In conclusion, we strongly suggest that researchers employing the mAb blocking assay using anti-γδTCR mAb perform γδTc alone controls in parallel, to quantify the extent of effector cell death occurring as a result of mAb treatment. This cell death can then be taken into account and subtracted from the difference observed in cytotoxicity upon application of this mAb, which should give a more accurate value for the extent to which true receptor blocking impairs cytotoxicity. IL-2 may be excluded from cytotoxicity assays to minimize the deadly synergy observed upon B1 stimulation, keeping in mind that this may reduce overall cytotoxicity and thus perhaps should be tested for each target cell line in advance. Previous work using B1 or B1.1 for blocking, ours included, should be reevaluated in this light. Perhaps the γδTCR is not as involved in target killing as we thought.

## Ethics Statement

This study was carried out in accordance with the recommendations of the Research Ethics Guidelines, Health Research Ethics Board of Alberta—Cancer Committee with written informed consent from all subjects. All subjects gave written informed consent in accordance with the Declaration of Helsinki. The protocol was approved by the Health Research Ethics Board of Alberta—Cancer Committee.

## Author Contributions

All the authors contributed to research design. ID and GS conducted experiments and performed data analysis. GS wrote the manuscript.

## Conflict of Interest Statement

The authors declare that the research was conducted in the absence of any commercial or financial relationships that could be construed as a potential conflict of interest.

## Abbreviations

αβTc, alpha beta T cells; AICD, activation-induced cell death; AnnV, annexin V; CalAM, calcein AM; FB, FACS buffer; FBS, fetal bovine serum; IL, interleukin; mAb, monoclonal antibodies; γδTc, gamma delta T cells; γδTCR, gamma delta T cell antigen receptor; PBMCs, peripheral blood mononuclear cells; SSC, side scatter; ZA, Zombie Aqua.
