# Supplementary material for: Apoptosis Induced via Gamma Delta T Cell Antigen Receptor “Blocking” Antibodies: A Cautionary Tale
Source: Front Immunol. 2017 Jun 30;8:776. doi: 10.3389/fimmu.2017.00776 (PMC5492911; doi:10.3389/fimmu.2017.00776)
Supplement: Supplementary file 1 [file Data_Sheet_1.pdf]

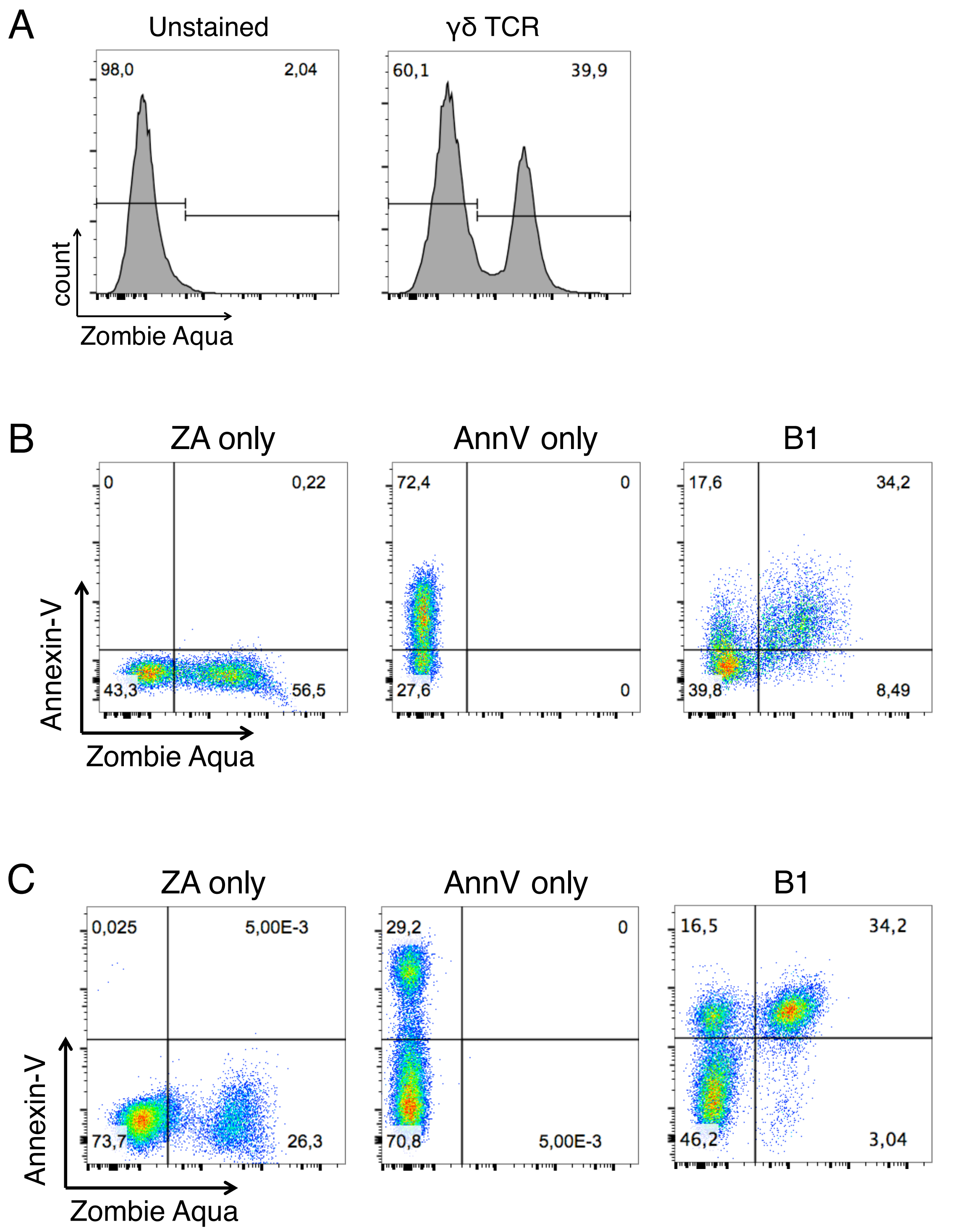

Figure S1. Gating controls for flow cytometry experiments. A. Donor 2 day 14 γδ T cells were untreated and unstained (left) or incubated with 1 µg anti-γδTCR antibody for 4.5 hours and stained with Zombie Aqua viability dye (gating for Fig. 1B). **B.** Donor 3 day 21 γδ T cells were treated with 1 µg anti-γδTCR antibody for 4.5 hours and stained with Zombie Aqua and Annexin V FITC. Single stained controls for Zombie Aqua (left, ZA only) and Annexin V (middle, AnnV only) were used to set gates for the experiment shown in Fig. 1C. **C.** Gating for Fig. 2A "no IL-2" samples shown here.

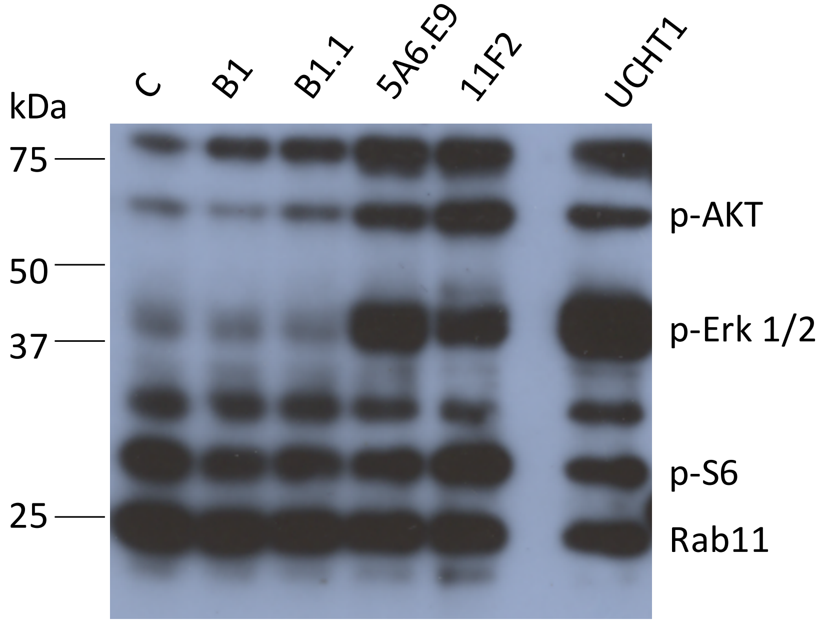

Figure S2. 5A6.E9 and 11F2 anti-γδ TCR antibody clones induce γδ T cell activation. Day 20 donor 9 γδ T cells were stimulated with 5 μg of the indicated antibodies for 1 min. Lysates were run on 12% SDS-PAGE gels, transferred to PVDF membranes and probed with the PathScan Multiplex Western Cocktail I (Cell Signaling Technology) to detect the indicated signaling proteins. Shown here is a representative example from 3 independent experiments.

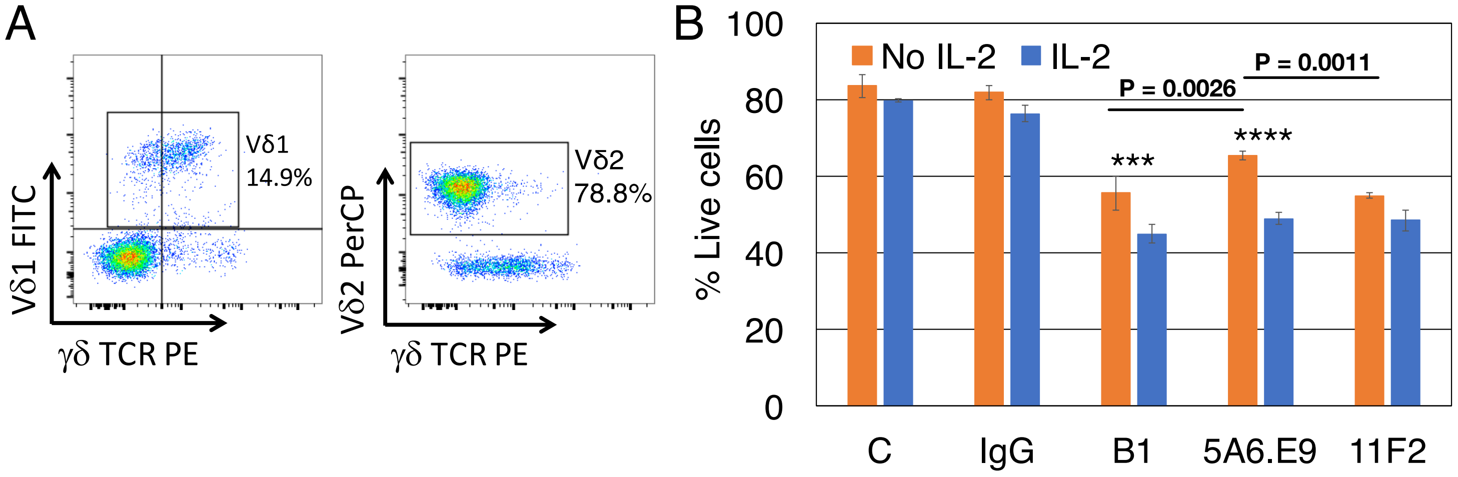

Figure S3. A γδ T cell culture with Vδ2 predominance is sensitive to apoptosis induced by all three anti-TCRγδ antibody clones tested. A. Day 19 donor culture 4-1 was stained with anti-TCR Vδ1 FITC, anti-TCR Vδ2 PerCP, and anti-TCRγδ PE, then analyzed by flow cytometry. Plots of Vδ1 FITC and Vδ2 PerCP versus anti-TCRγδ PE are shown**. B**. Day 19 donor culture 4-1 cells were incubated without (C = control) or with the indicated antibodies in the presence or absence of IL-2 for 4.5 hours, stained with Zombie Aqua (ZA) viability dye and AnnexinV-FITC, then acquired by flow cytometry. % Live cells were both ZA- and AnnexinV-negative. Two way ANOVA followed by Bonferroni's multiple comparisons tests were performed and identified highly significant differences between all antibody-treated and IgG-treated cells with and without IL-2 (not indicated, $P < 0.0001$). Significant differences in γδ T cell viability for each antibody treatment with or without IL-2 are indicated (*** $P < 0.001$, **** $P < 0.0001$). P values are shown for comparison of 5A6.E9 with B1 and 11F2 in the absence of IL-2. No significant differences were observed among antibody treatments in the presence of IL-2.

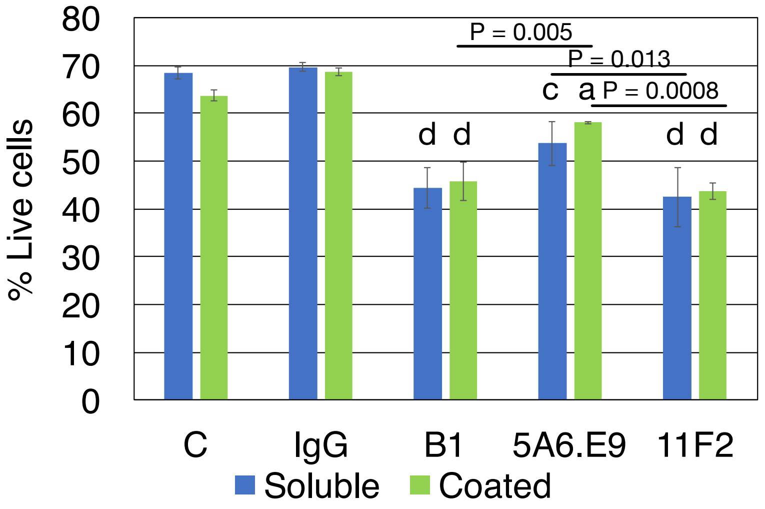

Figure S4. There is no difference in viability of cells treated with soluble versus immobilized anti-TCRγδ antibody clones. No differences in viability of Donor 8 culture 2 day 18 cells were induced by soluble versus immobilized (coated) B1, 5A6.E9 or 11F2 antibody treatments; however, significant decreases in viability were observed comparing antibody-treated- to IgG-treated cells. Two way ANOVA followed by Bonferroni's multiple comparisons tests were performed to identify significant differences between antibody-treated and IgG-treated cells (a, $P < 0.05$; c, $P < 0.001$; d, $P < 0.0001$) or among antibody treatments (line indicates groups compared and P values are given).

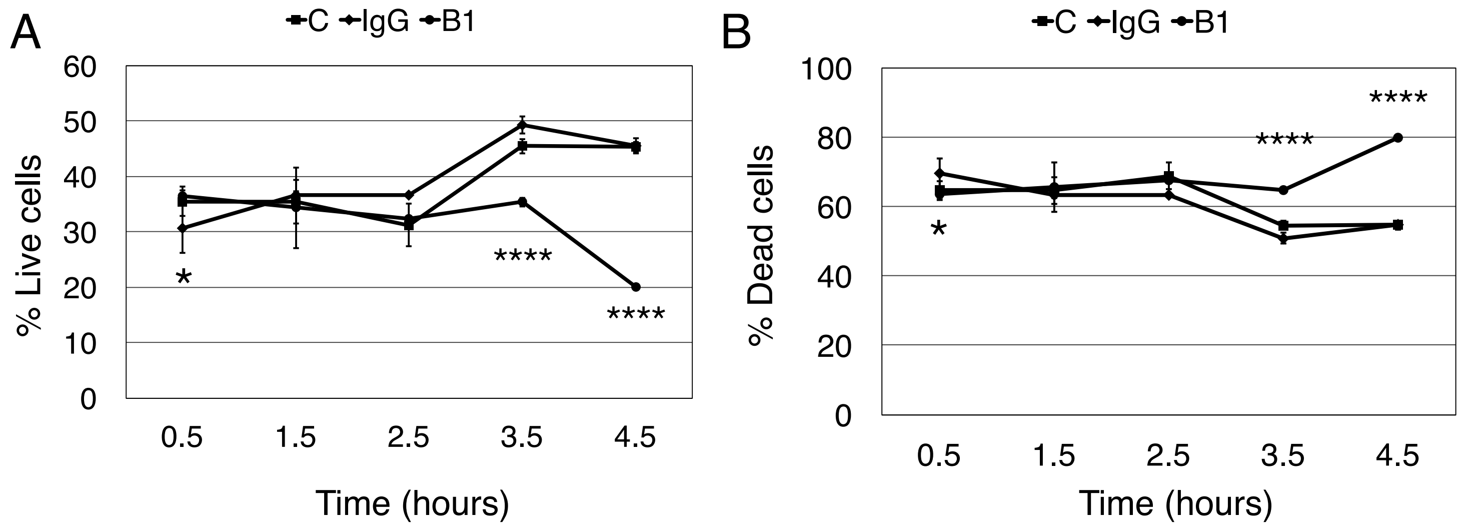

Figure S5. Kinetics of apoptosis induction by B1 antibody. A. 600,000 donor 8 day 17 γδ T cells were untreated (C) or incubated with 1 µg IgG control or B1 anti-γδTCR antibody, as indicated, for 30 minutes in 100 µl medium for 30 minutes, then an additional 100 µl medium was added (t=0 hours). Cells were further incubated for 1 - 4 hours and stained with Zombie Aqua viability dye and Annexin V FITC. Live cells were Zombie Aqua and Annexin V-negative. **B.** For the experiment described in A, the sum of early apoptotic, late apoptotic and necrotic cells make up the dead cell fractions shown here. Two way ANOVA followed by Bonferroni's multiple comparisons tests were performed to identify significant differences between B1-treated and IgG-treated cells at the indicated time points (* $P < 0.05$, **** $P < 0.0001$).

ID	Day	% Vδ1	% Vδ2	% γδTCR+Vδ1-Vδ2-	% Purity	Figure(s)
1-1	21	49.1	32.2	8.7	90.0	1A,E,F; 2H,I; 3B
1-2	20	70.0	14.9	11.5	96.4	2A-G; S1C
2-1	14	38.1	51.6	5.5	95.2	1B; S1A
3-1	21	23.2	58.3	3.5	85.0	1C,D; S1B
3-2	19	28.7	50.8	6.7	86.2	1F; 2D-G; 3B
4-1	19	14.9	78.8	4.2	97.9	1F; 2D-G,I; 3A,B; S3A,B
4-2	18	14.8	67.8	2.4	85.0	4C,D
5-1	22	40.3	49.0	6.2	95.5	2I; 3B
6-1	19	43.4	42.1	11.1	96.6	3C-F
7-1	19	39.1	16.1	11.0	66.2	4A-D
7-2	19	2.1	89.1	1.1	92.2	3D,F
8-1	19	27.1	49.0	5.5	81.6	4C,D
8-2	19	77.9	6.2	12.0	96.1	2I-K; 3D,F; S4; S5A,B
9-1	21	11.1	77.1	3.8	92.0	S2

Table S1. γδ T cell subset percentages and purities for donor cultures. Donor cultures were stained with Zombie Aqua and then with antibodies recognizing pan γδ TCR, Vδ1 TCR and Vδ2 TCR on the indicated day. Purity is the sum of %Vδ1, %Vδ2 and %γδTCR+Vδ1-Vδ2-. The figures in which results were obtained using these cultures are listed.
